# Determining the perception of stigma of individuals with ostomies

**DOI:** 10.1186/s40359-025-03112-1

**Published:** 2025-07-10

**Authors:** Azize Karahan, Ebru Akgün Çıtak, Banu Çevik, Aysel Abbasoğlu, Ziyafet Uğurlu

**Affiliations:** Department of Nursing, Faculty of Health Science, Başkent University, Baglıca Kampüsü Fatih Sultan Mahallesi Eskişehir Yolu 18.km TR, 06790 Etimesgut, Ankara, Turkey

**Keywords:** Colostomy, Ileostomy, Quality of life, Ostomy, Stigmatization, Stoma

## Abstract

**Purpose:**

The aim of the study is to determine the stigmatization perceived by individuals with intestinal ostomy to recognize these issues and assist them in the adjustment to having an ostomy.

**Methods:**

The study is descriptive and qualitative study. Interviews were conducted with a total of 14 individuals who were able to communicate, and had a permanent intestinal ostomy in the outpatient center of a university hospital. The content analysis method was used in data analysis.

**Results:**

Three main themes and seven subthemes were identified. The main themes were “perceived stigma”, “changes in life” and “emotional responses”. The majority of the patients experienced internalized stigma.

**Conclusion:**

In this study, individuals with intestinal ostomies perceveid both internalized and experienced stigma. Negative feelings with perceived stigma restricted social lives, decreased relationships with relatives, neighbors, and friends, reduced the desire to go out and negatively affected body image and sexual life. Education programs can be organized to help them cope with this situation.

**Supplementary Information:**

The online version contains supplementary material available at 10.1186/s40359-025-03112-1.

## Introductıon

Stigma is an important problem that negatively affects individuals. It is defined as “discriminating one person from others, degrading one person from other people, seeing them in the community” [1]. Stigmatization, on the other hand, is the whole of the behavior of society towards some patient groups as a result of the prejudices and the exclusion of them from society [2, 3]. Physical differences can also lead to stigma [4, 5]. It is emphasized that individuals with an ostomy can also be exposed to stigma and internalize the stigma [5]. Internalized stigma is the acceptance of negative stereotypes in society with an individual withdrawing from the society with negative feelings such as worthlessness and shame [6]. As a result of the internalized stigma, people suffering from the disease may exhibit deviation behaviors, decrease their job performance, and even experience unemployment problems and social adjustment problems [7, 8]. One of the groups at risk in terms of stigma is individuals who have an ostomy. Although ostomy is a necessary treatment for some conditions, it can affect individuals physically, psychologically, socially, and spiritually. Conditions such as ostomy pouch leakage in an unwanted place or time can cause both individuals to be excluded by society and to make individuals feel uncomfortable, embarrassed, and guilty [8–10].

Ceylan and Vural [9]. conducted a qualitative study with 19 patients who have an ostomy, individuals reported that they were embarrassed by the presence of an ostomy due to the effects on odor, flatulence, and visual body changes. In a study comparing patients with temporary (*n* = 42) and permanent ostomy (*n* = 23); patients with permanent ostomies have been found to have more feelings of guilt and embarrassment than those with temporary ostomies [10].

In a qualitative study conducted with seven patients who had temporary ostomy; the patients stated that they were concerned about disclosing their ostomy and becoming stigmatized [11]. Qin et al. [12] reported that the level of stigma in Chinese patients with temporary ostomies is moderate. More over, ostomy patients experience stigmatizing sentiments from their medical staff before and after surgery [13].

Nurses should be aware of the concept of stigma in individuals with an ostomy, strengthen individuals ‘coping skills, support family members’ acceptance, raise awareness of other patients ‘and individuals’ behavior and attitudes, and protect individuals with ostomy from these behaviors and attitudes [8]. The individual with a ostomy should be supported by health personnel with problem-oriented coping strategies [11, 14, 15]. Raising awareness about the stigma is the first step to prevent it. Stigma may differ in social and cultural dimensions. However, there are no studies that directly address the concept of stigma in individuals with an ostomy in Turkish society.

In this study, it is aimed to determine the stigmatization perceived by individuals with intestinal ostomy and to reveal the problems and effects they experience. It is thought that the data will contribute to the development of solutions for the problems experienced by patients with stigma and to prevent negative effects such as social isolation that may be experienced due to stigma, work-life that make life meaningful, social relations, spirituality, and the individual’s distancing from the effort to heal.

### Research questions

1. What is the society’s atittudes towards individuals with intestsinal ostomy according to individual’s perception?
2. How does stigma affect individuals with intestinal ostomy?

## Method

### Participants and procedure

This descriptive and qualitative study was conducted in the outpatient center of a university hospital between July 2018-December 2018. Purposeful sampling was used for the selection of a convenient sample to determine the early and late postoperative ostomy perceptions by selecting individuals. Interviews were conducted with the participants who had been at least 2 months after the procedure. Individuals who underwent intestinal ostomy were included in the sampling. The patients were in contact with the WOC nurse before and after the procedure. According to hospital procedure individuals with intestinal ostomy are followed up in the polyclinic in the first week, 15th day, 3rd month, 6th month, and 12th month after discharge and are evaluated by the WOC nurse in process. If there is a complication in terms of ostomy after the first year, they can apply to the WOC nurse.

Individuals with intestinal ostomy who were followed up in the outpatient center of the university hospital where the study was conducted constituted the sample of the study with the purposeful sampling method. The appointment schedule of the outpatient center was daily obtained and participants who met the sampling criteria were identified and the interview was conducted after obtaining informed consent, interviews were conducted with a total of 14 individuals who were able to communicate, and had a permanent intestinal ostomy, and the interviews continued until reaching a saturation point. Saturation point means the collection of qualitative data to the point where a sense of closure is attained because new data yield redundant information. Data saturation is reached when no new analytical information arises anymore, and the study provides maximum information on the phenomenon.

### Ethics

The Medical and Health Sciences Research Board and Ethics Committee of the university where the study was conducted approved the study. Each participant was informed about the study and their permissions for the interviews were obtained. In addition, participants who agreed to participate in the study were asked to read and sign a consent form allowing interviews, access to medical records, and audio recording.

### Measure

The data was collected after verbal and written consent was obtained from the institution and patients. In-depth individual interviews were conducted with patients to determine the stigmatization perceived by individuals with an ostomy and to reveal their problems and effects of this issue. Data from the interviews were obtained using “*The questionnaire form related to demographic data form”* and *“Semi-structured interview form”. The* aim of the study was explained to the patients participating in the study and permission was obtained to use the audio recording device in the interview. The interviews were conducted in a quiet room where the patient’s comfort and privacy was ensured in the outpatient center of the hospital. The interviews were conducted by a female researcher. A voice recorder was used with the permission of the participants. Another female nurse researcher set the tape recorder in a position where the voice of the moderator and the patient could be heard sufficiently, she also followed and recorded the patient’s reactions. Before the interviews were terminated, the received notes from the researcher were reviewed with the participant. Interviews lasted approximately 25–45 minutes, the average duration was 35 minutes.

#### The questionnaire form related to demographic data form

The form consists of 13 questions directed at determining the sociodemographic features and illness features. Questions about sociodemographic characteristics include; age, gender, marital status, etc.

#### Semi-structured interview form

Open-ended questions (Box1 Semi-structured interview form in suplementary file) enabled to express individuals with ostomy thoughts freely and tell their experience by providing as many details and their lives and examples as they liked [16]. The aim of the interviews was explained to participants at the beginning by moderators. Questions were determined according to the aim of the study and the literature. The answers of these questions were explained under the data collection section.

### Data analysis

The data recorded on the tape recorder was listened to firstly and transcribed verbatim. The analysis of the data was analyzed by two researchers separately, then they were discussed with other researchers and finalized. The content analysis method was used in data analysis. The following steps were followed for content analysis [16].

1. *Coding the data*: The researchers read the data set several times and repeatedly worked on the resulting codes.
2. *Determination of the themes*: Codes were brought together and examined and tried to find common aspects.
3. *Organizing codes and themes*: The determining themes and codes are arranged.
4. Findings were interpreted after they were presented.

To ensure the validity and credibility of this study, data were analyzed by two different researchers independently, after comparison of the interpretations, the themes and categories were finalized after the discussion of researchers to find the proper interpretation. Consistency was enhanced by the confirmation of the content analysis by three peer researchers outside of the research team who were experienced in the field of qualitative research [17, 18]. The Consolidated Criteria for Reporting Qualitative Research -COREQ guidelines were followed in reporting the methods.

## Results

Most of the participants were female (10 female) in the study and all are married. The average age of the participants was 61.28 ± 9.87 (min:45, max:80). Two of the patients were working, 7 patients retired one patient was unemployed and four patients were housewives for a total of 14. All patients were married. Nine of the patients were diagnosed with colon cancer and 5 of them with ovarian cancer diagnosis. Twelve of these patients had a colostomy and 2 had an ileostomy. The range of time since surgery was 2 months to 7 years have passed since ostomy surgery. Spouses of 7 patients, daughters of 3 patients, 3 patients themselves, and a caregiver of one patient provided ostomy stoma care. Information on the patients participating in the study is included in Table 1.

**Table 1:** Demographic features of participants, *N* = 14

Characteristics	Number	Percent (%)
**Mean age**: 61.28 ± 9.87 (min:45, max:80)		
**Gender**		
Female Male	10 4	71.4 28.6
**Education Status**		
Elementary school High school University	5 3 6	35.7 21.4 42.9
**Marital status**		
Married	14	100
**Diagnose**		
Colon Cancer Ovarian Cancer	9 5	64.3 35.7
**Duration of ostomy**: 22.4 ± 4.21 (min: 2, max:84) months		
**Occupation**		
Unemployed Employed Retired Housewife	1 2 7 4	7.1 14.3 50 28.6

**Fig. 1:**
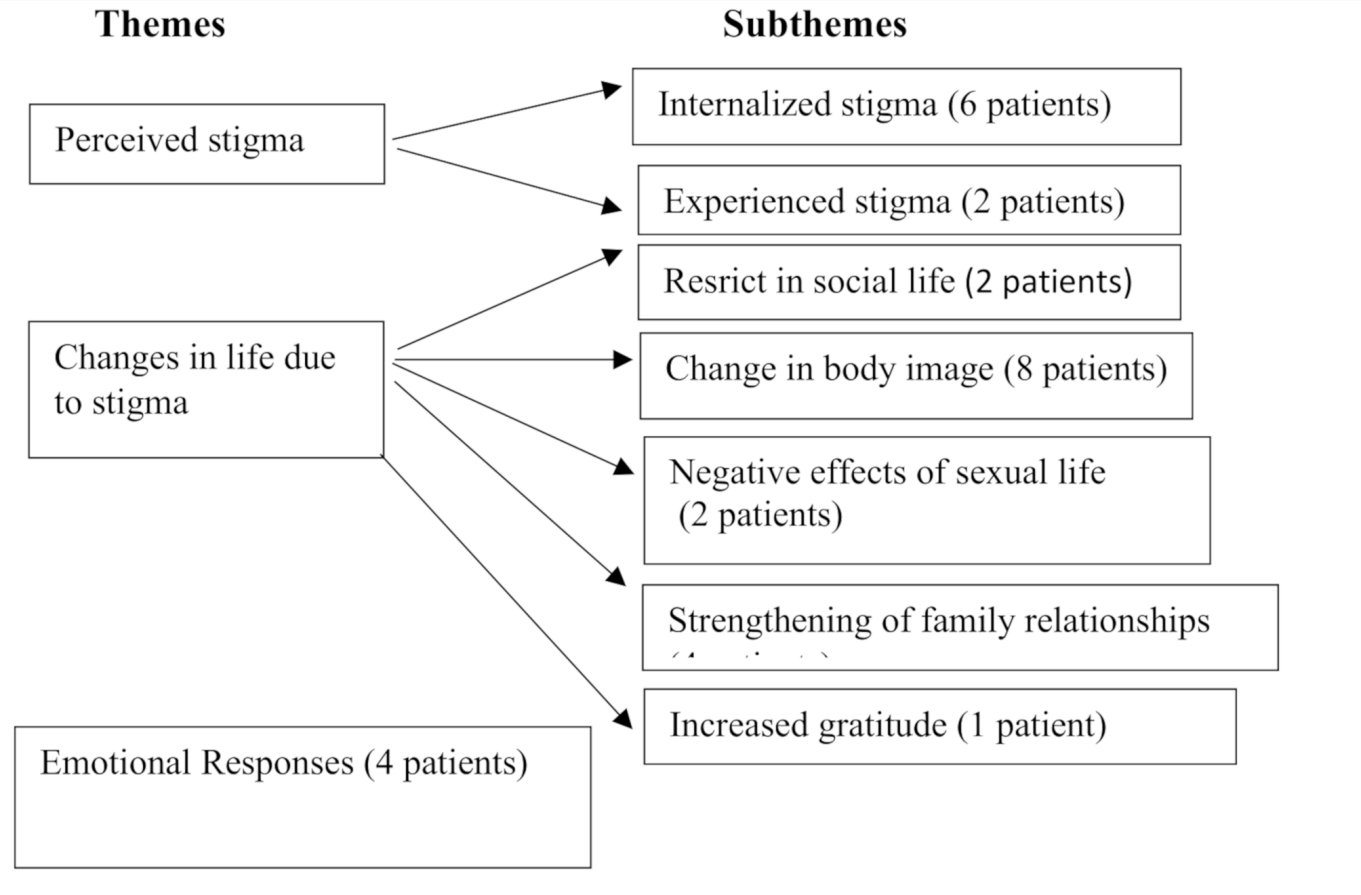
Themes and subthemes

Three main themes emerged as “perceived stigma”, “changes in life” and “emotional responses” in the study. Seven sub-themes have been identified. The themes and subthemes were presented in Fig. 1. (Figure I show Themes and subthemes)

### Main theme one: perceived stigma

In the study, internalized stigma and experienced stigma sub-themes were determined in the stigma theme perceived by the expressions of individuals. Patients experience more internalized stigma perception. Eight patients (7 females, 1 male) in the study did not tell anyone about their disease and colostomies as a result of internalized stigma and isolated themselves from their social circles. Quotes from participants are as follows:

“….…*I isolated myself after I got sick. I’m not going out……. I didn’t tell anyone when I had surgery*,* my neighbors have not known surgery*.” (P10, Female, 65 years old, 2 months after surgery).

Another patient’s expression is that *I can’t go to everyone*,* I can’t use everyone’s toilet”* (P 12, Female, 61 years old, 36 months after surgery).

“*Nobody knows from my neighbors or friends. Only my mother and my sister know from my family. I spend only 5 minutes a day with my neighbors”.* (P 11, Female 41 years old, 5 months after surgery).

*“I said that it smells so badly to my friends ‘they say*,* there is no odor*,* we did not feel like you. But*,* I know*,* it smells.”* (P12, Female, 61 years old, 36 months after surgery).

In the interviews, three patients expressed the stigma experienced. Patient statements about the experienced stigma are given below;

“…*I went to the village and visit my friend. I used to toilet. My friend knew about my illness. After I used to the toilet*,* she said her daughter to you to check the toilet after me. I heard this sentence. I never go to the toilet in another place”. (*While the patient said these sentences, she seems very sad). (P11, Female,41 years old, 5 months after surgery).

*“We discharge the colostomy bag in the toilet and bathroom. Because its smell disturbs my daughter. My daughter coughs because of the smell*,* so I empty my colostomy bag myself*”. (P6, female, 80 years old, 5 months after surgery)

*“One of the participants told the stigma she experienced: ‘When my intestinal ostomy was opened*,* I heard that people talked about me*,* they said was she going to be like that? I never communicated to the people I heard this conversation again.”* (P12, female, 61 years old, 36 months after surgery).

### Main theme two: changes in life-related to stigma

In the study, participants stated that the stigma due to ostomy caused changes in their lives. Five sub-themes were identified sub-theme of changes in life associated with stigma. These sub-themes are “restriction in social life”, “change in body image” and “negative effects of sexual life” “strengthening of the family”, and “increased gratitude” In this study, all of the participants stated that they could not participate in a social environment due to uncontrolled stool leakage and gas releasing from their ostomies. As an example of the restriction sub-theme in social life is given below.

*“……. When I feel the gas releasing if I’m with my friend*,* I’m going to the bathroom right away. Therefore*,* when I visit my friends*,* I do not spend too much time. I tried managed like this*.” (P4, male, 65 years old, 30 months after surgery).

“…….*I think I will no longer work*. *Before I got sick*,* I would be involved in many social activities. I played table tennis. Now that I have a ostomy*,* I think I will not be able to do these activities*. (P 11, female, 47 years old, 5 months after surgery).

In the study, we determined that body images were adversely affected after opening the intestinal ostomy. In particular, more than half of women (eight women) stated that they had difficulty choosing clothes due to the ostomy and they had to choose bigger size clothing to hide their ostomy so that it wouldn’t be noticed. Examples that can be given to the sub-theme of change in body image;

*“When I wear thin clothes in the summer season*,* the ostomy bag suddenly swells.” I run to the bathroom and empty the ostomy bag. I wear trousers two sizes larger. When people look behind my pants*,* they think that the pants belong to someone else*. (P14, Female, 63 years old, 12 months after surgery).

Considering the statements of the participants, ostomy also negatively affected the sexual lives of them. They had difficulties talking about their sexual life. Almost all of the participants indicated that they have no sex life. In particular, their internalized stigma reflected negatively on their sexual life due to a negative perception of the ostomy on body images.

“*We have no sexual relations with my husband.* (She was crying when she was saying this sentence, P9, female 63 years old, 84 months after surgery).

“…*There is a coldness in sexual life. Sometimes*,* we have a sexual life*,* but nothing between us for a long time”*. (P7, Female, 45 years old, 17 months after surgery).

In addition to the negative effects of the ostomy, some participants have also stated the positive effects of an intestinal ostomy on their life.

The four participants-stated that they received much support from their friends and relatives in addition to their families. They also indicated that their family relationships are getting stronger and they are happy because of this situation. As an example of strengthening family relationships;

*“My husband does everything for my comfort. Neighbors know but they never ask. My husband and children are very interested in me.”* (P9, female 63 years old, 84 months after surgery).

*“Stoma saved my life*,* I take it natural. I do my own stoma care. My family and friends were positive about stoma.”* (P2, Male, 72 years old, 3 months after surgery).

In our study, patients also frequently expressed the spiritual dimension related to the ostomy. Participants stated that they should be grateful for their current situation. An example of gratitude is that a patient stated a sentence as follows.

“*I prayed to God in my world. I gratitude my god. If this happens to me it will be better for me (P2*,* male*,* 72 years old*, 3 months after surgery*).*

### Main theme three: emotional responses

The last theme is emotional reactions. It has been determined that participants experience emotions such as anxiety, sadness, embarrassment, restlessness, and anxiety due to stigma. Some of the patient statements about the feels experienced are given below.

“*I cry and go out in the bathroom…. I am not looking at the mirrors*,* I’m angry with my image in the mirrors” (P7*,* female*,*45 years old*, 17 months after surgery*)*.

*‘I was worried when I first saw the ostomy bag” (The patient was crying*,* P7*,* female*,* 45 years old*, 17 months after surgery*).*

*“I went to an official institution that day. while there*,* there was an audible gas release from my intestines. I couldn’t say anything to the people around me.* (P1, Male, 54 years old, 12 months after surgery).

## Dıscussıon

This study was performed to determine the stigmatization in individuals with an ostomy. We founded that patients experienced both internalized and experienced stigma. While some of the participants explained, their relatives supported them very much, their fears such as stoma-related gas releasing and stool leakage caused isolation from society and concealed their intestinal ostomy from many people. The majority of participants stated that they left many activities in their social life after the intestinal ostomy was opened.

Individuals with ostomy face many psychosocial problems in their lives related to intestinal ostomy. These problems include depression, anxiety, low body image, lack of self-confidence, decreased social interaction, denial, loneliness, hopelessness, and unhappiness [19–21]. Individuals with an intestinal ostomy are faced with problems due to stigmatization such as odor, leakage, restrictions in clothing (preferring loose clothing), travel restrictions, disease-related shame, disgust, and perceived social stigma [20–22]. One of the themes we determined in our study is “life changes related to stigma”. Sub-themes of this theme are changes in body image, negative effects of sexual life, and restrictions in social life. The sub-themes of our study are similar to the results of the study in the literature. In studies, the biggest concerns of patients with ostomy were reported that the fear of being notified of their ostomies by others, odor, and releasing noisy gas from the intestinal [20, 22]. In our study, individuals stated that the ostomy bag is noticed under their clothes (especially when the bag is filled with gas) and they prefer plenty of clothes to hide. A qualitative study, individuals with ostomy stated that their ostomy bags restricted their choice of clothing and that they had difficulties, especially in summer [23]. In a study conducted by Aktaş and Göçmen Baykara [24]to determine the effects of a ostomy on patients and their partners, 60% of patients with ostomy experienced psychosocial problems such as anxiety, avoiding sexual life, depression, low self-esteem, avoiding social activities, and not meeting with friends [24].

Individuals with ostomy worry about being stigmatized by the community for reasons such as the ostomy pouching system being visable, odor, and uncontrolled gas release and leakage. Impaired body image affects this perception more negatively. This situation may be caused by the thoughts of the individuals not to be accepted by society and perceives themselves differently from society due to changes in their lives. Therefore, individuals with an intestinal ostomy can avoid going to public places such as cinemas, restaurants, sports or recreation facilities, or interacting with their friends [19, 20, 25, 26]. In this case, while individuals restrict their social activities, dealing with the ostomy adjustment process and the problems caused by the ostomy becomes difficult [20]. In our study, the participants experienced internalized and experienced stigma depending on their intestinal ostomy. More than half of the participants stated that they did not tell their ostomies to anyone except their first degree relatives, they limited their social activities, they stayed a very short time in places where they had to go, and they did not go to other people’s toilet. In a study with 209 patients who have ostomy in China, 44% of experienced high levels of stigma, with some characteristics such as low ostomy acceptance by one’s spouse or other family members, poor perceived body image, stool leakage, and no experience of participating in activities with other ostomy patients [8].

In our study, participants stated that ostomy also had positive effects such as strengthening family ties and increasing their gratitude behavior. In the literature, it is emphasized that family and social support are very important in terms of improving self-esteem and reintegration with the social environment of individuals with a ostomy (9,26). In our study, the importance of family support was emphasized by individuals with an intestinal ostomy in the literature. The participants stated that their children, spouses, and close relatives gave a lot of support during ostomy surgery, postoperative, and discharge process. However, it was determined that the participants restrict themselves from social activities due to the effects of the intestinal ostomy, despite the support of their family friends.

In our study, another positive effect of the intestinal ostomy is the increase in gratitude behavior. The participants stated that they were grateful for their current situation. This positive mood is very valuable for individuals to deal with the negative effects of intestinal ostomy. Popek et al. [27] reported that patients with colostomy had optimistic and positive emotions and their quality of life was high. On the other hand, Gautam et al. [28] determined in a study with 130 patients with ostomies in Nepal that adherence to ostomy was negatively affected in patients who perceived no family support.

Another theme is “emotional responses” related to stigma. In our study, patients reported that they experienced emotions such as anxiety, sadness, embarrassment, restlessness, and anxiety due to stigma. Individuals can experience positive and negative emotions together depending on their ostomy. Individuals with ostomy often feel sad and disabled [19, 20, 24]. Also, individuals feel unhappy, self-disgusted, embarrassed [19, 20]. In addition to the literature on psychosocial problems seen in individuals with stoma, Ayaz-Alkaya [19] stated that individuals with stoma experience anxiety, depression and stigmatization after their stoma is created and their body image is adversely affected. Smith et al. [5] determined that disgust reaction plays an important role in stigmatization and there is a negative correlation between the feeling of disgust and compliance in patients with a ostomy (*n* = 195) and a positive correlation between stigmatization sensations. In a qualitative study on individuals with an ostomy, they did not talk to anyone other than their families about their ostomies due to their embarrassment, and even some of them did not tell their children because they did not dislike the smell of the ostomy [9]. Similarly, a multicenter study found that stigma negatively affected psychosocial adjustment to the stoma [28].

### Limitations and future directions

In this study, most participants were women and, perioperative periods of paritipants differed between each other. During the study period, those who applied to the outpatient center of the university hospital and those who agreed to participate in the study were predominantly women. In order to determine the effect of ostomy in terms of gender, it may be recommended to conduct studies that include male and female samples equally.

WOC nurses follow individuals up who have an ostomy, as a hospital procedure more closely in the first year, in case of complications in the following years. It was thought that this situation might affect the results of the study.

In this study, the time elapsed after ostomy surgery was not limited in order to determine the participants’ exposure to stigma more comprehensively and how they perceived this situation. In future studies, sample selection can performed from individuals with similar stoma surgery time to determine the effect of the time elapsed after ostomy surgery.

## Conclusıons

In this study, it was aimed to determine the perceived stigmatization related to the intestinal ostomy, experienced problems related to the issue and their effects. We determined that negative feelings such as anxiety, sadness, and embarrassment related to perceived internalized and experienced stigma restricted social lives, decreased relations with relatives, neighbors, and friends, reduced the desire to go out, and negatively affected body image and sexual life. Being aware of the stigma perceptions of individuals with an intestinal ostomy as healthcare personnel, providing the appropriate conditions for individuals to express themselves; supporting and directing individuals, and their relatives in this regard will contribute to improving the quality of life of individuals. It may be recommended to conduct studies that included avereness and interventions educational programs to deal with the perception of the internal and external stigma of the patients.

## Electronic supplementary material

Below is the link to the electronic supplementary material.

Supplementary Material 1

## Data Availability

No datasets were generated or analysed during the current study.

## Abbreviations

WOC: Wound, Ostomy and Continence
