# Supplementary material for: Determining the perception of stigma of individuals with ostomies
Source: BMC Psychol. 2025 Jul 10;13:767. doi: 10.1186/s40359-025-03112-1 (PMC12247216; doi:10.1186/s40359-025-03112-1)
Supplement: Supplementary file 1 — Supplementary Material 1 [file 40359_2025_3112_MOESM1_ESM.docx]

**Box 1: Semi-structured interview form**

| 1. What does it mean to you to be an individual with a ostomy?  2. How was your life affected after having ostomy?  3. How did your ostomy affect your relationships in social life?  4. How do you evaluate the attitudes of your family, close circle, and other people in the community towards ostomy?  5. How did the attitudes of your family, close circle, and other people in the community affect your life? |
| --- |
